# New strategies and perspectives on managing IgA nephropathy

**DOI:** 10.1007/s10157-019-01700-1

**Published:** 2019-02-13

**Authors:** Haresh Selvaskandan, Chee Kay Cheung, Masahiro Muto, Jonathan Barratt

**Affiliations:** Department of Infection, Immunity and Inflammation, University of Leicester, Leicester, LE1 9HN UK

**Keywords:** IgA nephropathy, Berger’s disease, Novel treatments, Repurposed therapy

## Abstract

IgA nephropathy is an inflammatory renal disease characterised by the deposition of IgA in the glomerular mesangium and is the most commonly reported primary glomerulonephritis worldwide. Thirty to forty percent of patients with the disease develop progressive renal function decline, requiring renal replacement therapy within two decades of diagnosis. Despite this, accurate individual risk stratification at diagnosis and predicting treatment response remains a challenge. Furthermore, there are currently no disease specific treatments currently licensed to treat the condition due to long standing challenges in the nature and prevalence of the disease. Despite this, there have been exciting recent advances in the field that may represent paradigm shifts in the way IgA nephropathy is managed in the near future. In this review, we explore the evidence base informing current approaches to management and explore new strategies and future directions in the diagnosis and management of IgA nephropathy.

## Introduction

IgA nephropathy (IgAN) is an inflammatory renal disease characterised by the deposition of IgA in the glomerular mesangium [1]. Described first by Jean Berger in 1968, it is now recognised as the most commonly reported primary glomerulonephritis worldwide [2–6]. Despite 50 years of study revealing valuable insights into its pathophysiology [7, 8], there are still key features that remain unexplained; IgAN has a distinct geographical variation, being more common and aggressive in East and South East Asia, and only 30–40% of patients develop a progressive form of the disease, typically necessitating renal replacement therapy within two decades of diagnosis [9, 10]. Given that the incidence of IgAN peaks among young adults, understanding why progression occurs is an issue of socioeconomic importance. Accurate individual risk stratification at diagnosis and predicting treatment response also remain a challenge.

The variation in prevalence and progression of IgAN present barriers to establishing standardised, evidence-based approaches to treatment. Robust trials have traditionally been limited by low recruitment, given the relative rarity of IgAN among Caucasians, and there has been a dearth of globally recruiting clinical research trials. Despite this, there have been some exciting advances which address these issues, including the development of international disease registries [11], non-invasive biomarkers [12–14], and an increase in interest in running international trials for novel and repurposed targeted therapies in IgAN. In this review we provide an overview of the evidence informing current management approaches, and then explore future directions relating to the diagnosis and treatment of IgAN.

## Current approaches

The diagnosis of IgAN relies on histological examination of a renal biopsy, which will demonstrate predominant IgA deposition in the renal mesangium [15]. At diagnosis a constellation of clinicopathological parameters have been shown to provide some insight into the risk of progression. These include estimated glomerular filtration rate (eGFR), amount of proteinuria, blood pressure, and renal histology [16]. Management typically involves supportive interventions followed in some cases by immunosuppression and/or tonsillectomy; although there is marked heterogeneity in the approaches adopted internationally, likely fuelled by regional variations in the nature of IgAN and a paucity of robust clinical trials [17]. Here we provide an overview of the evidence behind current approaches to managing IgAN, and compare key points from Kidney Disease Improving Global Outcomes (KDIGO) and Japanese guidelines [16, 18].

### Supportive management

#### Diet modification

Low-protein diets reduce glomerular hydraulic pressures and have been reported to slow renal functional loss [19]. A Cochrane review of low-protein diets concluded that limiting protein intake reduced the occurrence of RRT or death by 31% [20]. There is, however, no specific evidence for their use in IgAN and this strategy is rarely employed. In contrast, low-sodium diets have been shown to be efficacious in IgAN. The benefits of sodium restriction are thought to be related to sodium sensitivity of blood pressure, which correlates with renal ultrastructural damage [21]. A small cross-over trial showed that low-sodium diets reduced proteinuria even in normotensive IgAN patients [21], however it was not clear if renin–angiotensin system (RAS) blockade was optimised prior to commencing a low-salt diet. The deleterious effects of heightened sodium sensitivity are thought to be mediated through the renin–angiotensin system (RAS) and indeed, sodium restriction enhances the antiproteinuric effects of RAS inhibition in IgAN [22].

#### Weight optimisation

Body mass index (BMI) at either extreme correlates with increased morbidity and mortality in CKD [23–25]. A Chinese case–control study demonstrated an association between low BMI (< 18.5 kg/m^2^) and likelihood of end-stage renal disease (ESRD) in IgAN [26], and a similar Japanese study found patients with elevated BMIs (> 23 kg/m^2^) had lower rates of proteinuria remission following treatment [27]. Obesity is postulated to increase proteinuria by inducing ultrastructural changes to the glomerular basement membrane [28, 29]. While these correlations are likely to have been confounded by factors such as hypertension [30, 31], a small RCT nevertheless has demonstrated benefits of weight loss in overweight patients diagnosed with primary IgAN, beyond maximal RAS blockade, hypertension control and protein/sodium restriction [32].

#### Smoking cessation

There is some evidence from case–control and observational cohort studies that smoking may be an independent and dose-dependent risk factor for progressive renal function decline in IgAN [33–35].

#### Fish oil supplementation

KDIGO and Japanese guidelines both suggest use of fish oil in IgAN, with KDIGO limiting its recommendation to patients with persistent proteinuria despite optimised supportive care [16, 18]. The evidence for benefit from fish oil is, however, mixed with a collection of small RCTs showing fish oil can slow renal function loss, but with mixed effects on proteinuria [36–38]. Perhaps more compelling are the meta-analyses of all fish oil data which have failed to demonstrate any consistent benefit in IgAN [39]. As such most nephrologists do not advocate fish oil in IgAN.

#### Renin–angiotensin system blockade

The role of RAS blockade in IgAN patients is well established. Both guidelines recommend RAS antagonism with an angiotensin-converting enzyme inhibitor (ACE-I) or an angiotensin II-receptor blocker (ARB) in patients with proteinuria greater than 1 g/day. Several RCTs have demonstrated the benefits of RAS blockade [40–42], and a meta-analysis of 11 such trials found ACE-I/ARBs were successful at reducing proteinuria, lowering blood pressure and slowing the rate of renal function decline. This held true even when 6 of the 11 studies that were deemed ‘poor quality’ (assessed by the Jadad score) were removed from the analysis [43]. Indeed, this finding has been corroborated by more recent studies, such as the STOP-IgAN trial, in which the use of ACE-I /ARBs in addition to other supportive measures was as effective as combination therapy with immunosuppressants in preventing decline in eGFR in IgAN [44]. RAS blockade is the first-line management in IgAN. Dual RAS blockade is controversial. While dual therapy has been shown to result in a greater reduction in proteinuria compared to monotherapy, this occurs at the cost of more frequent adverse events with no clear evidence for improvement in long-term eGFR [45, 46].

### Tonsillectomy

The evidence for tonsillectomy varies worldwide, and the practice is more common in Asia compared to Europe. It is thought to reduce upper airway infections and consequently the production of poorly *O-*galactosylated IgA1 (galactose deficient or Gd-IgA1) [47–50]. Studies from Asian cohorts report a reduction in proteinuria following tonsillectomy [51–54], however many of these were uncontrolled retrospective studies, and favourable outcomes were mostly seen in association with corticosteroids. Conversely, large retrospective European studies have found no benefit with tonsillectomy [55–57]. This difference may highlight ethnic variations in the pathogenic drivers for IgAN. Alternatively, the difference may be due to differences in the time point at which tonsillectomy is performed; the procedure is often performed soon after diagnosis in Japan, while in Europe it is employed only following recurrent episodes of tonsillitis. The KDIGO guidelines does not recommend tonsillectomy in IgAN, while the Japanese guidelines suggest its use early in the natural history of the disease irrespective of whether there is a history of tonsillitis. Both guidelines acknowledge the need for a well constructed RCT of tonsillectomy in IgAN.

### Immunosuppression

A number of traditional immunosuppressive agents have been tried in IgAN, including corticosteroids, cyclophosphamide, azathioprine and mycophenolate mofetil. Much of the available evidence for efficacy of immunosuppression in IgAN is, however, weak and based on a small number of studies that do not reflect current standard of care for proteinuric glomerular disease [58–60]. These studies have several limitations [61], they notably lacked a ‘run in’ phase making it unclear if a benefit would have existed beyond optimised supportive therapy. Both guidelines do, however, suggest a treatment course of systemic glucocorticoids in those with proteinuria above 1 g/day and eGFR higher than 50 ml/min/1.73 m^2^ (KDIGO) or 60 ml/min/1.73 m^2^ (Japanese Guidelines) despite supportive care.

The role of corticosteroids in IgAN remains an area of contention, and practice varies considerably worldwide. The STOP-IgAN trial, conducted in Germany, included a 6-month run-in period, over which time patients were given intensive supportive management. Patients were then randomised to receive either immunosuppressive therapy (corticosteroids or cyclophosphamide and corticosteroids followed with azathioprine) or to continue on supportive management and were followed up for 3 years [44]. The STOP-IgAN study demonstrated little benefit of immunosuppressive agents over supportive therapy [62, 63] and importantly 34% of the patients in the run-in phase had proteinuria which settled to below 0.75 g/day (the inclusion criteria for the subsequent randomisation stage), demonstrating the value of supportive management. Corticosteroids were associated with a higher rate of clinical remission compared to supportive therapy alone, however, this came at the expense of more adverse events, including glucose metabolism disturbances, weight gain and infections. A substudy of the retrospective VALIGA cohort did report benefit with corticosteroids in patients with an eGFR < 50 ml/min compared to a propensity matched cohort that did not receive corticosteroid monotherapy, although no comment on safety could be made as no adverse event data had been collected [64].

The TESTING trial, which recruited the majority of patients from China, compared methylprednisolone (0.8 mg/kg/day; maximum 48 mg/day) against placebo, and demonstrated that a relatively high corticosteroid dose had potential renal benefit, with a reduction of patients in the methylprednisolone group reaching the primary composite renal outcome (40% decrease in eGFR, ESRD or death due to kidney failure; 5.9% vs 15.9% in the placebo group), and a reduced mean annual eGFR decline in this group (− 1.79 in the methylprednisolone group vs − 6.95 ml/min/1.73 m^2^ in the placebo group). However, this came at a cost with a significantly increased rate of adverse events, and the study was terminated early by the data safety monitoring committee due to the increased number of life-threatening infections in the treatment arm [65]. Of interest, the rate of annual eGFR decline in the placebo arm of the TESTING study was much higher compared to the supportive care arm of the STOP-IgAN study (− 6.95 vs − 1.6 ml/min/1.73 m^2^), supporting findings from a previous observational study that patients of East Asian origin may have more rapid rates of renal decline in IgAN [66], and therefore could conceivably respond differently to immunosuppressive therapy. There are clearly differences in clinical practice and use of corticosteroids in IgAN worldwide, with prednisolone commonly used in Japan at an average dose of 10–15 mg/d (i.e. much lower than the original TESTING study). Further study of the effects of lower dose corticosteroids on renal decline in IgAN vs their associated adverse effects is required in the context of well-designed randomised controlled trials, and the currently recruiting TESTING low dose study (ClinicalTrials.gov Identifier: NCT01560052), comparing 0.4 mg/kg/day methylprednisolone to placebo, should hopefully provide some answers.

In 2018 the role of immunosuppression in IgAN remains unclear [67, 68]. There is no convincing evidence for the value of MMF, cyclophosphamide and azathioprine in IgAN. The current paucity of data to support the use of traditional immunosuppressive agents in IgAN highlights a need for development of novel therapies to treat this important cause of kidney disease. Over the past 5 years there has been a slowly increasing number of clinical trials testing novel and repurposed therapies in IgAN and it is an exciting time in the field of IgAN. Here, we summarise recent trials and their outcomes.

## Novel therapies

### Atacicept (NCT02808429) and blisibimod (NCT02062684)

**Fig. 1:**
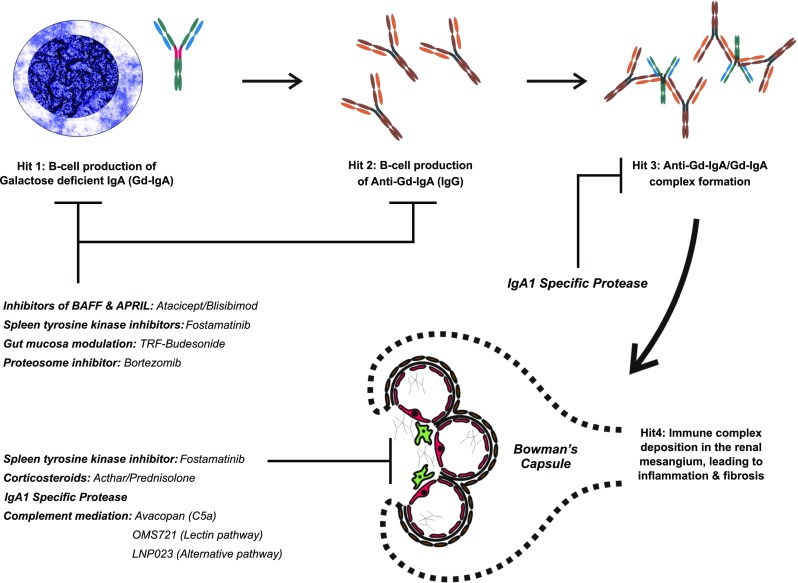
Targets of novel/repurposed drugs. Target 1: B-lymphocyte activation results in the production IgA1 poorly *O-*glycosylated at the hinge region (gd-IgA1, highlighted in red). This results in the generation of auto-antibodies, which leads to the formation of immune complexes. The deposition of immune complexes in the mesangium will lead to varying degrees of inflammation and fibrosis. *Atacicept, blisibimod, TRF-budesonide, bortezomib* and *microbiome modulation* can modulate B-lymphocyte activity, which may theoretically lead to a reduction in the production of Gd-IgA1, the auto-antibodies and circulating immune complexes. Target 2: *Acthar, avacopan, OMS721 and LNP023* all act in the kidney to suppress local inflammation and subsequent fibrosis, dampening the downstream consequences of immune complex deposition. *Fostamatinib* acts both at the level of the B-cell and directly in the kidney reducing the inflammatory response to IgA deposition. *IgA1 proteases* are capable of digesting both circulating and deposited IgA and IgA immune complexes

BAFF (B-cell activating factor) and April (a proliferation inducing ligand) are members of the tumour necrosis factor family which mediate B-cell function and survival [69]. Over expression of BAFF in transgenic mice has been shown to result in IgA deposition in the renal mesangium. BAFF and April levels are elevated in the serum of IgAN patients and correlate with disease activity [70–73]. TACI (transmembrane activator and calcium-modulator and cyclophilin ligand interactor) binds BAFF and April, mediating their downstream effects through the NF-κB pathway. Atacicept is a fusion protein containing the extracellular ligand binding domain of TACI and can therefore block the downstream effects of BAFF and April (Fig. 1). Blisibimod is a selective antagonist of BAFF. Atacicept and blisibimod have been investigated in other autoimmune conditions, including SLE and rheumatoid arthritis (RA) [74, 75]. Phase II studies to assess safety and efficacy in IgAN are underway (Atacicept) or have recently finished (blisibimod) and results are awaited (Table 1).

**Table 1:** Clinical trials of novel/repurposed drugs in IgAN for which results are awaited

Trial	Intervention	Inclusion criteria	Exclusion criteria	Trial design	Primary end point	Follow-up duration
Atacicept NCT02808429	Atacicept at varying doses vs placebo	Proteinuria 1–6 g/day Stabilised on RASi for 8 weeks	Prior cyclophosphamide treatment Use of other immunosuppressants within 4 months	Randomised, double-blind, placebo-controlled Phase II trial	Incidence of adverse events	180 weeks
BRIGHT-SC NCT02062684	Blisibimod vs placebo	Proteinuria 1–6 g/day Stabilised on RASi for 8 weeks	Immunosuppressant use over last 6 months or corticosteroid use over last 3 months. Malignancy over last 5 years	Randomised, double-blind, placebo-controlled Phase II/III trial	Reduction of proteinuria at 24 weeks	104 weeks
SIGN NCT02112838	Fostamatinib at varying doses vs placebo	Stabilised on RASi for 90 days. BP < 130/80 Proteinuria > 1 g/day at diagnosis and > 0.5 g/day at second screening visit	Recent use of corticosteroids, cyclophosphamide, mycophenolate mofetil, azathioprine or rituximab	Randomised, multicentre, double-blind, placebo-controlled, Phase II trial	Reduction of proteinuria at 24 weeks	24 weeks
VELCADE NCT01103778	Bortezomib	Proteinuria > 1 g/day Stabilised on RASi for 4 weeks	Peripheral neuropathy, history of cardiac problems, malignancy within last 3 years	Open-label, Phase IV trial	Reduction of proteinuria at 1 year	1 year
ACTHAR NCT02282930	Acthar gel	Proteinuria > 1 g/day Stabilised on RASi for 3 months BP > 130/80 HSP patients included	Crohn’s disease or celiac sprue Glucocorticoid treatment in last 3 months Immunosuppressive therapy in last 6 months Previous ACTH treatment History of malignancy History of cardiac or pulmonary disease	Open-label, Phase III trial	Reduction in proteinuria at 1 year, stabilisation of eGFR at 1 year	1 year
OMS721 NCT02682407	OMS721 vs placebo	Patients on immunosuppressive patients included, if on stable dose for 2 months Optimised RASi, BP < 150/90, Urine ACR > 600 mg/g	Renal transplant History of malignancy Use of belimumab, rituximab, or eculizumab within last 6 months HSP within 2 years	Randomised, double-blind, placebo-controlled, Phase II trial	Incidence of adverse events	18 weeks
LNP023 NCT03373461	LNP023 vs placebo	Stabilised on RASi for 90 days eGFR ≥ 30, proteinuria ≥ 0.75 g/day	Recent use of immunosuppression, history of drug/alcohol abuse, malignancy	Randomised, double-blind, placebo-controlled Phase IIa/IIb trial	Reduction of proteinuria at 90 days	180 Days

All RCTs required adult patients to have biopsy proven IgAN as part of their inclusion criteria, and excluded patients with secondary IgAN, liver disease, infections, and pregnant/breast feeding women. All trials, except SIGN trial, explicitly stated exclusion if evidence of significant glomerular/cortical scarring was present on biopsy

*RASi* renin–angiotensin system inhibition, *IgAV* IgA vasculitis/Henoch–Schonlein purpura

### Fostamatinib (NCT01738035)

Tyrosine kinase (TK) pathways have major roles in homeostasis and disease, and a number of TK inhibitors have been licensed for treatment of a variety of conditions [76]. Spleen tyrosine kinase (SYK) is a non-receptor TK that may modulate a number of key pathogenic pathways in IgAN [77]. SYK acts as a signal transducer following B-cell receptor activation, mediating downstream signalling and promoting B-cell maturation and survival. Additionally, there is mounting evidence to suggest that SYK plays a role in the kidney in IgAN. Stimulation of mesangial cells in vitro with IgA1 purified from IgAN patients triggers SYK phosphorylation, along with the release of pro-inflammatory mediators [77]. Furthermore, patients with endocapillary hypercellularity in their biopsy (a lesion which occurs in 20–50% of patients with IgAN and may signify amenability of the disease to treatment) exhibit higher renal SYK expression compared to patients without the lesion [78]. There is therefore a strong case for targeting the SYK pathway in IgAN. Fostamatinib is a selective SYK inhibitor that has been studied in RA where it lowered disease activity compared to placebo. However, this came at the expense of adverse effects at a rate of up to 72.2%, with the commonest being diarrhoea and hypertension. There were no deaths reported [79]. A Phase II trial of fostamatinib to evaluate its safety and efficacy in IgAN has recently finished (Table 1).

### Rituximab (NCT00498368)

Rituximab is a widely used monoclonal antibody which targets the CD20 receptor on B-cells. It had been postulated that rituximab could reduce Gd-IgA1 and anti-Gd-IgA1-IgG antibody production by causing B-cell depletion, which would in turn provide renoprotection [80]. However, a recent trial comparing rituximab with supportive care to supportive care alone, failed to show an effect of rituximab on Gd-IgA1/autoantibody levels, eGFR and proteinuria (Table 2).

**Table 2:** Clinical trials of novel/repurposed drugs in IgAN for which results have been published

Trial	Intervention	Inclusion criteria	Exclusion criteria	Trial design	Primary end point	Follow-up	Sample size	Outcome
Avacopan NCT02384317	Avacopan 30 mg twice daily	After 4 weeks of optimised RASi eGFR > 60 eGFR > 45 if decline < 10 mL/min/1.73 m^2^ over prior 24 weeks Urine PCR > 1 g/g	Proteinuria > 8 g/day, Malignancy within 5 years, cardiac disease, immunosuppression in last 24 weeks. HSP within last 2 years	Open-label Phase II trial	Incidence of adverse events	12 weeks	7	Reduction of proteinuria in 6 of 7 patients
Rituximab NCT00498368	Rituximab + supportive care vs supportive care alone	After 2 months of optimised RASi Proteinuria ≥ 1 g/day BP < 130/80 HSP included	eGFR < 30 > 6 months of steroids > 50% glomerular senescence or cortical scarring, history of Crohn’s disease or celiac sprue	Randomised, open-label, multicentre, Phase IV trial	Proteinuria and eGFR at 12 months	12 months	34, American	No effect on end points
NEFIGAN NCT01738035	TRF-budesonide 8 mg or 16 mg + supportive care vs supportive care alone	After 6 months of optimised RASi Urine PCR ≥ 0.5 g/g OR urine protein ≥ 0.75 g/24 h eGFR ≥ 45, BP ≤ 160/100	Immunosuppression over previous 24 months, or at any time for IgAN Renal transplant, diabetes, malignancy over last 3 years	Randomised, multicentre, double-blind, placebo-controlled, Phase II trial	Urine PCR at 9 months and 12 months	12 months	149, European	Reduction in urine PCR achieved by budesonide

All RCTs required adult patients to have biopsy proven IgAN as part of their inclusion criteria, and excluded patients with secondary IgAN, liver disease, infections, and pregnant/breast feeding women. The rituximab trial explicitly stated exclusion of if evidence of significant glomerular/cortical scarring was present on biopsy

*RASi* renin–angiotensin system inhibition

### TRF-budesonide (NCT01738035)

Targeted-release formulation of budesonide (TRF-budesonide) is designed to deliver budesonide to the distal ileum, a major site of mucosal B cell localisation within the mucosal associated lymphoid tissue (MALT). It has been long established that there is an as yet ill-defined link between the mucosal immune system and IgAN [81], and therefore targeting the gut MALT represents a novel strategy in the treatment of IgAN. As TRF-budesonide is heavily degraded by first pass metabolism in the liver, with only 10% entering systemic circulation, this formulation could significantly reduce the systemic adverse effects of corticosteroid therapy while suppressing mucosal B-cell activation and proliferation [82]. The NEFIGAN Phase IIb trial investigated the efficacy and safety of two doses of TRF-budesonide compared to placebo in IgAN patients already receiving maximal supportive care (Table 2). The study demonstrated a significant reduction in proteinuria after 9 months treatment with TRF-budesonide, and although more adverse events were noted with treatment, this did not reach statistical significance [81, 83]. While eGFR was stable in the treated group, there was a significant decline in the placebo treated group which was greater than expected for patients receiving optimised RAS inhibition, and commentators have questioned the robustness with which supportive therapy was administered overall [81]. It is, however, likely that the observed reduction in time averaged proteinuria seen with TRF-budesonide would likely translate to long-term improvements in renal outcome [84, 85]. This trial represents a positive first step in the search for a targeted therapy in IgAN and highlights the importance of the gut-kidney axis in IgAN. A Phase III registration study, NefIgArd, is now underway (NCT03643965).

### Bortezomib (NCT01103778)

Bortezomib is a semi-selective plasma cell proteasome inhibitor used in the treatment of multiple myeloma [86]. Proteasomes are essential intracellular protein complexes that degrade unneeded or damaged proteins by proteolysis [87]. Interferon gamma and alpha induce proteasomes to switch to immunoproteasomes, which have important roles in antigen processing and T cell activation [88]. Dysregulation of the proteasome:immunoproteasome axis has been shown in mononuclear cells in IgAN with over expression of the immunoproteasome, increased nuclear translocation of factors related to the NF-kB pathway, and more severe disease manifestations including increased proteinuria [89]. Use of bortezomib has expanded recently, with trials in solid tumours, amyloidosis, lymphomas and antibody mediated allograft rejection [90–92]. A clinical trial is currently underway to evaluate the safety and efficacy of bortezomib in IgAN (Table 1). A general concern is that the side effects of bortezomib (peripheral neuropathy, thrombocytopenia, rash, fatigue and anorexia) are likely to significantly limit its use in asymptomatic young patients with IgAN [90].

### Acthar (NCT01103778)

Adrenocorticotropic hormone (ACTH) is currently licensed in the United States of America for idiopathic nephrotic syndrome and nephrotic syndrome due to lupus nephritis. ACTH exerts its renoprotective effects though steroid-dependent and independent mechanisms, the latter thought to be mediated by the melanocortin 1 receptor (MC1R). MC1R is found on several kidney cell types, including glomerular endothelial cells, podocytes, mesangial cells and tubular epithelial cells. MC1R agonists have renoprotective effects in vitro, and reduce proteinuria in animal models [93, 94]. A recent multicentre retrospective case series of ACTH use in resistant nephrotic syndrome included five patients with IgAN. Of these five patients, three experienced a reduction in proteinuria of ≥ 30%, however one patient ended the treatment early due to weight gain and hypertension. Of the full set of 44 patients with a variety of glomerular pathologies, 13 reported adverse events including weight gain, hypertension, hyperglycaemia, hypokalaemia and upper respiratory tract infections [95]. It was not clear if this was dose dependent. An open-label single group trial is currently underway, with the goal of assessing the safety and efficacy of ACTHAR in IgAN (Table 1).

### Avacopan (NCT02384317), LNP023 (NCT03373461) and OMS721 (NCT02682407)

Since the first description of IgAN it has been clear that complement activation plays an important role in the final pathway to glomerular injury in IgAN [96, 97]. C5a is a potent local inflammatory mediator, and is generated through the cleavage of C5, following the activation of C3 convertase [98]. The presence of C5a in the kidney correlates with histological severity and proteinuria in IgAN [99]. Targeting C5a therefore offers an opportunity to suppress the local inflammation contributing to progressive renal disease, while preserving the formation of the C5b-9 (membrane attack complex) which plays a crucial role in the elimination of gram negative bacteria [100]. Avacopan, a C5a receptor blocker, has been evaluated in an open-label Phase II trial in seven patients with IgAN (Table 2). At the end of 12 weeks, proteinuria reduced in 6 of the 7 patients, with 3 of the 7 patients showing significant improvement to UPCR < 1 g/g. Longer term studies are needed in IgAN, however, there is an increasing literature on the safety and efficacy of avacopan in patients with ANCA-associated vasculitis, where it has been shown to be effective in replacing high-dose corticosteroids, although it has been associated with hepatic dysfunction and an increased risk of infection in a small proportion of patients [101].

Both the alternative and lectin pathways of complement activation are believed to be important in IgAN [102, 103]. The alternative pathway is an important amplification mechanism for classical and lectin-pathway activation, resulting in greater opsonisation and generation of the terminal lytic pathway. The two proteases Factor D and Factor B are essential for this tightly regulated amplification process. Selective small-molecule reversible inhibitors of Factors B and D have been developed and these inhibitors efficiently block alternative pathway activation. A Phase II Phase IIa/IIb dose ranging study of LNP023, a first in class oral inhibitor of Factor B, in IgAN has recently commenced with recruitment expected to be completed in early 2019 (Table 1).

Glomerular lectin pathway activation correlates with increased proteinuria and histological damage in IgAN [104]. Mannose-binding lectin associated serine protease 2 (MASP-2) is a key component of the lectin pathway and promotes C3 convertase formation and subsequent downstream inflammatory effects. Targeting MASP-2 may thus reduce glomerular lectin pathway activation while still allowing C3 convertase to be generated through the classical and alternative pathways. The MASP-2 inhibitor OMS721 is currently being evaluated in Phase II and Phase III studies in IgAN (Table 1).

### Experimental therapies for the future

#### IgA1 proteases

There has been longstanding interest in the use of bacterial proteases that cleave IgA1, as a potential therapy for IgAN [105]. IgA1 proteases are produced by bacteria including *Streptococcus pneumonia, Haemophilus influenzae*, and *Neisseria meningitides*, and directly cleave the hinge region of human IgA1, but not IgA2. Evaluation of this therapy has been hampered by the lack of an appropriate mouse model, as murine IgA exists as one isoform that more closely resembles IgA2 and lacks the extended hinge region of IgA1. More recently, a humanised mouse model, that expresses human IgA1 and develops spontaneous mesangial IgA deposition, has been used to show that administering recombinant IgA1-proteases from *H. influenzae* reduced IgA1 and C3 deposition, glomerular macrophage infiltration and fibronectin deposition, with a corresponding reduction in haematuria [106]. However, mesangial deposits recurred shortly after completion of the treatment course, and a strong IgG response against the IgA1-protease occurred after 3 injections, suggesting that this approach may need to be part of a combined strategy with other therapies. There have been no reported studies in human IgAN to date.

#### Modulation of the microbiome

There has been a great deal of interest in potential associations between the microbiome and human disease. The microbiome is defined as the collective genomes of the microbes (bacteria, bacteriophages, fungi, protozoa and viruses) within a microbiota (specific niche, such as the human gut) [107]. The number of microbes in the human body far exceeds the number of human cells by approximately ten to one, with the largest number of microbes residing in the intestinal tract. It is well established that around 30% of patients experience a flare of their IgAN, in terms of visible haematuria, in conjunction with a mucosal infection, most commonly an upper respiratory tract or gastrointestinal infection. In a transgenic mouse that overexpresses BAFF and develops hyper-IgA syndrome, spontaneous glomerular IgA deposition was dependent upon the presence of gut microbiota, as mice raised in germ-free conditions were protected until gut microbiota were introduced [73]. In a cross-sectional study, patients with progressive IgAN had a reduced faecal microbial diversity, compared to those with non-progressive IgAN and healthy subjects [108]. With the advent of mucosal targeted therapies, such as TRF-budesonide, which have demonstrated efficacy in IgAN there is a heightened interest in the possible contribution of the gut microbiota in the development of IgAN. Manipulating the gut microbiota with probiotics may prove to be a viable and low risk therapeutic strategy in the future.

#### Treatment of IgAN recurrence

Treatment of IgAN recurrence post renal transplantation is an extremely understudied area, and the optimum immunosuppressive strategy is not known. The approach to treatment is generally based on evidence in IgAN in native kidneys. ACEis/ARBs are recommended, however evidence for other treatments is lacking. It is hoped that once efficacy and safety of the new therapies being tested is confirmed there will be a desire to study these drugs in IgAN transplant recurrence.

#### IgA vasculitis

Another understudied disease is IgA vasculitis, a condition with strong similarities to IgAN. Current treatment recommendations are based largely on the guidelines for IgAN. It is again hoped that should any of the drugs currently being evaluated prove to be efficacious and safe in IgAN that there will be a drive to test them in IgA vasculitis.

## Conclusions

In 2018 the future looks bright with regard to potential new therapies to treat IgAN. The pharmaceutical industry has engaged with the IgAN community to unprecedented levels and global studies in IgAN are now becoming the standard. Including participants from different ethnic backgrounds is essential if we are to evaluate any new treatment as it is still unclear whether the underlying pathogenic pathways driving IgAN are the same in all parts of the world.
